# The Impact of Nationality on the Psychophysiological Stress Response and Academic Fulfilment in the Final Degree Dissertation

**DOI:** 10.3390/ijerph18084035

**Published:** 2021-04-12

**Authors:** Ana Ramírez-Adrados, Beatriz Martínez-Pascual, Cristina Gonzalez-de-Ramos, Silvia Fernández-Martínez, Valentín Emilio Fernández-Elías, Vicente Javier Clemente-Suárez

**Affiliations:** 1 Faculty of Sport Sciences, Universidad Europea de Madrid, Villaviciosa de Odón, 28670 Madrid, Spain; ana.ramirez@universidadeuropea.es (A.R.-A.); beatriz.martinez@universidadeuropea.es (B.M.-P.); CRISTINA.GONZALEZ2@universidadeuropea.es (C.G.-d.-R.); silvia.fernandez@universidadeuropea.es (S.F.-M.); VALENTIN.FERNANDEZ@universidadeuropea.es (V.E.F.-E.); 2 Grupo de Investigación en Cultura, Educación y Sociedad, Universidad de la Costa, Barranquilla 080007, Colombia

**Keywords:** autonomic modulation, multiculturalism, heart rate variability, stress, university, physiotherapy academic achievement

## Abstract

The aims of this study were: i. to analyze the effect of nationality on the psychophysiological stress response of physiotherapy last year students in their final degree dissertations; and ii. to analyze the relationship between the stress response and academic results according to nationality. We evaluated the autonomic stress response, cortical arousal, distress subjective perception, and objective and subjective academic fulfilment in Spanish, Italian, and French physiotherapy students during their final degree dissertation. Results showed a large anticipatory anxiety response before the dissertation in the three student groups. Only the Spanish group showed an increased tendency in the habituation process, reducing the psychophysiological stress response during the dissertation, while the Italian and French groups maintained a large sympathetic activation until the end of the dissertation. Cortical arousal and subjective perception of distress were similar in the three nationalities. In addition, no correlation between academic fulfilment and autonomic modulation was found. We concluded that there was no nationality effect in the psychophysiological stress response of physiotherapy last year students in their final degree dissertation, all of them showing a large anticipatory anxiety response.

## 1. Introduction

Higher education studies are marked by the implantation of the Bologna plan, which facilitates student mobility, to make higher education more inclusive and accessible, improving the intercultural and learning mobility of European students. The main aims of the Bologna, declaration in 1999, were a demand for a “greater compatibility and comparability of the systems of higher education” to enhance the “attractiveness” and “competitiveness” of Europe’s higher education systems, and foster the better employability of European graduates [1].

Since this inclusion, the multiculturalism in the higher education context has increased annually, and students from different nationalities coexist in the same classrooms [2]. In the same way, the finalization of degree studies requires the defense of a final degree dissertation, with an expert court as tribunal. For this final degree project students construct a synthesis of theory, develop a research methodology, select and apply appropriate research methods, and finally conduct a comprehensive analysis and a discussion [3].

Like other academic experiences, such as the objective structured clinical examination (OSCE) and clinical practices, the final degree dissertation is perceived as the single most demanding task at university, causing fear, inquietude, and uncertainty [4]. Using new technological devices, we can implement organic response evaluation instruments which provide us with direct information on the autonomous nervous system modulation or even cortical arousal of participants [5,6,7]. Previous studies [8,9,10,11,12] have monitored a large increase in sympathetic autonomic modulation in university students during stressful academic experiences, as well as an anticipatory anxiety response before academic events.

The control of the stress response is important for achieving academic performance, since the correct neuronal function of the prefrontal regions is necessary for the development of complex processes such as memory, decision-making, and the learning process. The stress response produces the activation of the hypothalamic–pituitary–adrenal axis, which releases chemical mediators that affect the correct neuronal functioning of this region, and therefore affect memory and learning [13,14]. In addition, it has been shown that stress and anxiety reduce the regional cerebral blood flow in the prefrontal cortex, with a premature and intense effect on the executive functions, therefore they constitute one of the main threats in educational contexts [15].

Different contextual parameters can modulate the stress response, such as nutrition, psychological profile, physical activity, as well as, nationality [16,17,18,19,20,21]. Overweightness and obesity are negatively related with academic performance, as well as the poorer aerobic fitness of students [22]. It was also identified how psychological inflexibility, loneliness, and resilience are psychological factor related to academic success [23]. All these parameters not only affect academic outcomes, but also could negatively affect the autonomic stress response of students, being able to limit their resources in important academic events, such as dissertations, exams, or clinical evaluations [20,24]. The importance of these parameters in the academic field is clear, and they not only affect the student; nutritional, physical, and psychological profile will also affect teachers, making them more sensitive to stress and burnout related symptoms [25,26]. Regarding nationality, differences were found in higher education students, Colombian presenting students a higher sympathetic activation than Spanish students in their clinical psychology stays [16]. Analyzing the effect of psychophysiological stress response in academic fulfilment among students from different nationalities would promote effective programs to improve students stress management in these academic events [27].

In higher education, and specifically in physiotherapy degree students, the effect of different countries of origin on final degree dissertation has not been previously studied; therefore, we proposed the present study with two main aims: i. to analyze the effect of nationality on the psychophysiological stress response of physiotherapy last year students in their final degree dissertations; and ii. to analyze the relationship between the stress response and academic results according to nationality. The principal hypothesis was that students´ psychophysiological stress response would be different depending on their nationality, and that students with a higher stress response would present lower academic results.

## 2. Materials and Methods

A total of 140 last year physiotherapy degree students (4th year) at a Spanish University, who had to defend their final degree dissertation were contacted by mail to invite them to participate in the study. On the day of the defense, 27 students refused to participate in the study and 3 records were not valid. Finally, we analyzed 110 students in the last year of their physiotherapy degree from different countries during their final degree defense.

Students were divided in three groups: 55 Spanish students (SP: 16 women and 39 men, between 22 and 39 years old, M = 24.84 ± 2.50); 19 Italian students (IT: 5 women and 14 men, between 23 and 43 years old, M = 29.37 ± 6.41) and 36 French students (FR: 27 women and 9 men, between 22 and 29 years old, M = 23.86 ± 1.38). All the participants filled an informed consent form in accordance with the Helsinki Declaration (as revised in Brazil, 2013) and approved by the University Ethical Committee. Before the beginning of the research the experimental procedures were informed to all participants, indicating the right to withdraw from the study at any time and informed consent was obtained from each participant. Participation in this study did not have any type of compensation.

Students had to defend their final degree project to three professor specialists in the area. The project defense lasted 20 min, composed of 10 min of dissertation and 10 min of discussion with the professors. The defense was conducted in a conference room of the European University of Madrid (Spain).

We analyzed four different moments during the final degree dissertation: M1: 15 min before the dissertation; M2: first 1/5 of the dissertation; M3: last 1/5 of the dissertation; M4: 15 min after the end of the dissertation. A Polar V800 watch recorded the information, so it was not necessary to disrupt the dissertation to complete the assessments.

Autonomic modulation was measured in the four moments, while subjective perception of distress, cortical arousal, and expected academic achievement were measured in M1 and M4. In addition, the written final thesis defense achievement (0–10), the oral final thesis defense achievement (0–10), the written + oral final thesis defense achievement (0–10), and the mean academic achievement during the degree (0–10) were also obtained after the dissertation.

We analyzed the autonomic modulation of the participants by heart rate variability (HRV). These variables were monitored by the analysis of R-R interval of the heartbeat with a Polar V800 heart rate monitor (Polar, Kempele, Finland), as in previous research [28]. This is a non-invasive system that did not interfere in the normal activity of the participants, being composed of a chest band and a watch. The R-R series was analyzed using Kubios HRV software (version 3.0, Biosignal Analysis and Medical Imaging Group, University of Kuopio, Kuopio, Finland). The following HRV variables were evaluated: minimum heart rate (HRmin); mean heart rate (HRmean); maximum heart rate (HRmax); the square root of the average of the sum of the differences squared between normal adjacent R-R intervals (RMSSD); percentage of differences between normal adjacent R-R intervals greater than 50 ms (PNN50); ratio between low and high frequency band (LF/HF); the low-frequency band in normalized units (LFn); the high-frequency band in normalized units (HFn); and the sensitivity of the short-term variability (SD1) and the long-term variability (SD2) of the non-linear spectrum of the HRV.

A scale of subjective distress units (SUDS) was used to measure the subjective perception of distress, with scores between 0 and 100. Each of which represents a level of distress perceived by the subject at the time of valuation, ranging from zero (0), which implies “completely indifferent and cold; does not affect me” to one hundred (100), which means “so distressed and tense that I can’t deal with it”. This scale provides information based on the level of stress assessed by the individual, and which represents the cognitive relationship between the objective occurrence and the emotional response [16].

Cortical arousal was measured by the critical flicker fusion threshold (CFFT) (Lafayette, IN, USA) with a Lafayette Instrument Flicker Fusion Control Unit model 12,021. In the viewing chamber were simultaneously presented two light-emitting diodes (58 cd/m2), one for the left eye and one for the right eye. The stimuli were separated by 2.75 cm (center to center) with a stimulus to eye distance of 15 cm and a viewing angle of 1.9°. The inside of the viewing chamber was painted flat black to minimize reflection. The flicker frequency increment increased from 20 to 100 Hz until the student perceived fusion. Students had to respond by pressing a button upon identifying the fusion (ascending frequency) threshold [29]. Before the test, students performed practice trials to get accustomed with the CFF test. Three ascending trials were carried out. In each one, time was quantified as the amount of time that a student took for detecting the changes in the lights from the beginning of the test until the moment of pressing a button [14,29,30]. We used the critical flicker fusion threshold (CFFT) as it has been widely used in different contexts, like sports, military, education, and pharmacy, to evaluate cortical arousal and central fatigue [6,7,14,19,20,29,31].

Data are presented as mean ± standard deviation (SD). The sample calculation with a 99% of confidence level, 5% margin of error for this population was 110 participants. Normal distribution of data was checked using Shapiro–Wilk test. HRV, SUDS, and CFFT data were analyzed using mixed factorial ANOVA, which included the between-subjects factor of nationality and the within-subjects factor of time. Participant differences in objective and subjective academic achievements were analyzed using one-way ANOVA. After a significant F-value (Greenhouse–Geisser correction for the assumption of sphericity), differences between means were identified by using Bonferroni post-hoc procedure. To analyze correlations between the different study variables, we used the Pearson correlation test. The level of significance was set at *p* ≤ 0.05. Data analysis was performed using SPSS software v. 24 (IBM, Chicago, IL, USA).

## 3. Results

During the final degree defense project, we found a significant increase in PNN50 in M2 and M3 in Spanish students compared to Italian and French students (Table 1). No other significant differences were found in any HRV variable between the different nationalities.

SUDS and CFFT were similar in the students of different nationality before and after the final defense (Table 2). Regarding academic achievements, the written and oral final degree achievements were significantly higher in Spanish students than French ones (*p* < 0.05), and there was a tendency to be higher (ANOVA *p* = 0.063, post-hoc *p* = 0.046) (Table 3) in the total final degree thesis achievement. Italian students presented no significant differences in academic achievement compared with Spanish and French students. Moreover, the mean academic achievement during the whole physiotherapy degree was similar among the three nationalities. However, the expected achievement was similar in the three groups of students (Table 4). No correlation was found between academic achievement and any of the HRV variables and cortical arousal.

## 4. Discussion

The aims of this study were: i. to analyze the effect of nationality on the psychophysiological stress response of physiotherapy last year students in their final degree dissertations; and ii. to analyze the relationship between the stress response and academic results according to nationality. The initial hypotheses were not confirmed since no differences in the psychophysiological stress response and academic results of physiotherapy last year students from different nationalities were found.

The three national student groups analyzed presented low values of PNN50, RMSSD, HF, SD1, and SD2 in the moment before the final degree dissertation, showing a large anticipatory anxiety response [32]. This anticipatory anxiety response has been previously reported in high-risk environments, such as in high-performance sports or military maneuvers [14,33,34] and in some academic contexts, like clinical stays of physical therapy [8] and nursing students [9], objective structured clinical examination and simulation training in psychology students [10,11], and the laboratory practice of pharmacy and biotechnology students [19]. These unpredictable and uncontrollable situations are associated with a physiological stress response (high sympathetic modulation) that prepares individuals to respond to any possible occurrence. According to our results, nationality seems not to affect this anticipatory response, showing the same sympathetic hyperactivation in students of different nationality, in line with previous studies in university students [16].

Analyzing the habituation process during the final degree dissertation, we found an increased tendency in PNN50 from M2 to M3 in the Spanish group, a fact related with an increase in parasympathetic modulation. Nevertheless, Italian and French students maintained a high sympathetic response during the whole defense. This insufficiency habituation could be due to nationality differences that have a connection for example with the alertness level shown in each social and cultural environment, since previous studies have shown how some cultural elements, such as school location, could affect the stress and anxiety response [21]. Other study areas such as psychology found Colombian students in a clinical simulation scenario presented a higher parasympathetic modulation than Spanish students. This response showed how the daily context of where the student lives, is an autonomic modulator to consider for preparing efficient academic scenarios in higher education environments [16].

The analysis of HRV is a useful tool to analyze autonomic modulation. Previous authors have reported different sensibilities in HRV parameters to identify autonomic response. For example, the HRV analyses most sensible for analyzing the autonomic response of psychology students in OSCE were the frequency domain (LF and HF) and the nonlinear domain (SD1 and SD2), while the time domain (RMSSD and PNN50) showed a low sensibility [11]. However, other authors found a high sensibility in all HRV parameters in physiotherapy students [35]. Autonomic modulation has also been studied in a military environment, where the frequency domain (LF and HF) was more sensible to analyze changes in the sympathetic nervous system [13,14,34]. These differences may be due to the different exposition contexts and the different training and experiences of the subjects analyzed; factors that must be controlled to select the most sensible HRV parameters [10,36].

Regarding the subjective stress perception, the three student groups presented large SUDS values before the final degree dissertation, with no differences between nationalities. These high values in the stress perception are in line with the anticipatory stress response analyzed with the HRV parameters, showing a concordance between subjective and objective stress related variables. However, after the final degree defense the subjective stress perception decreased, although the sympathetic response remained. This response shows how independently of the decrease of perceived threat (decrease of SUDS), the sympathetic autonomic modulation presents a longer latency period, probably related to the original function of this defense system, which protected us against environmental stressors [37]. Other studies have shown a similar decrease in SUDS values when the stressor stimuli disappear, such as in experimented military parachute jumpers [38] or in clinical simulations in psychology students [10].

Cortical arousal was maintained during the final degree defense in the different nationalities analyzed. This could be explained by the duration of the final degree defense, not being long enough to cause central nervous system fatigue [31]. However, the results showed an increased tendency after the defense that was related to a decreased cortical arousal and information processing [29]. Previous researchers analyzed cortical arousal response in other highly stressful contexts, such as parachute jumps, combat situations, and ultra-endurance mountain races, showing how these contexts produce a decrease in cortical arousal; a fact interpreted as a symptom of central nervous system fatigue [36,38,39]. It seems that the duration of the stimuli, its intensity, and the threat perception can modulate the cortical response, showing how in the present research the final degree defense was a stimulus that, due to its characteristics of duration, intensity, and threat, did not cause a negative effect at a cortical level.

The subjective and objective academic achievement results were similar in the three student groups, not presenting a significant correlation with the HRV variables and CFFT analyzed. However, written and oral final degree project achievements were significantly higher in Spanish and Italian students compared to the French group. When we compared the mean academic achievement for the whole physiotherapy degree, no differences between the three groups were found, showing how a well-defined academic model can be implemented equally, regardless of nationality. In this line, the relationship between autonomic stress response variables and academic fulfilment has been analyzed in other research [11,16,21,35], showing no significant correlations between HRV parameters and academic achievement, in line with this research.

### 4.1. Limitations of the Study and Future Research Lines

The principal limitation of this study was the non-control of stress hormones (cortisol and alpha amylase) that would provide relevant information about the hormonal stress response and the habituation process.

The number of participants in each group was not homogeneous. The Spanish group (55 students) was larger than the French (36 students) or Italian (19 students) groups. The number between female students (48 students) and males (62 students) was not also equal. Future lines of research could analyze the autonomic modulation response in other stressful academic situations and in other degrees, as well as analyze the effect of the implementation of biofeedback equipment to improve the control of the stress response by students.

### 4.2. Practical Application

Final degree dissertations in physiotherapy students produce a large anticipatory anxiety response. The knowledge of the students´ autonomic modulation could be useful to prepare them during the degree by doing more practical sessions like the final degree defense. The stress produced by an unknown and important academic act like this would be reduced by increasing their habituation, due to greater exposure to simulated environments. The inclusion of relaxation techniques, which allow students to control their stress response in these important academic events, can also be recommended.

## 5. Conclusions

Physiotherapy last year students presented in their final degree dissertation a large anticipatory anxiety response independently of their nationality (Spanish, Italian, and French), and characterized by a high sympathetic autonomous modulation, maintained during the entire thesis defense. Nevertheless, cortical arousal was not negatively affected, and academic achievement showed no significant difference between groups.

## Figures and Tables

**Table 1:** Changes (Mean ± SD) in the autonomic stress response in the final thesis defense according to different nationality.

Variable	Nationality	M1	M2	M3	M4	F-Value	*p*-Value	Post Hoc
HRmin (bpm)	SP	78.54 ± 2.83	105.68 ± 4.70	87.97 ± 2.92	78.38 ± 2.58	0.62	0.646	
	IT	73.33 ± 2.45	103.63 ± 4.70	86.59 ± 3.31	77.45 ± 3.09			
	FR	71.98 ± 2.90	98.62 ± 4.41	85.74 ± 2.48	75.67 ± 2.37			
HRmax (bpm)	SP	147.59 ± 5.41	151.35 ± 7.31	128.51 ± 4.37	135.99 ± 4.94	0.849	0.506	
	IT	147.43 ± 4.72	141.82 ± 5.22	123.07 ± 5.11	121.78 ± 3.98			
	FR	150.64 ± 6.13	141.03 ± 4.66	124.22 ± 3.55	127.93 ± 3.12			
HRmed (bpm)	SP	109.68 ± 4.30	130.85 ± 4.20	111.28 ± 3.97	103.87 ± 3.78	0.65	0.618	
	IT	102.99 ± 3.88	128.29 ± 4.30	107.11 ± 4.72	100.75 ± 3.53			
	FR	103.02 ± 3.43	122.45 ± 4.55	105.18 ± 3.68	97.99 ± 3.45			
RMSSD (ms)	SP	38.95 ± 8.61	24.01 ± 6.74	26.95 ± 3.22	42.06 ± 7.67	0.88	0.469	
	IT	33.38 ± 6.49	12.39 ± 1.94	19.13 ± 3.15	31.27 ± 5.03			
	FR	41.32 ± 6.73	23.99 ± 5.37	22.06 ± 2.25	29.94 ± 2.02			
PNN50 (%)	SP	9.10 ± 2.45	24.01 ± 6.74	26.95 ± 3.22	9.18 ± 1.99	12.78	<0.000	M2: 1 > 2 (0.012) 1 > 3 (0.029)/M3: 1 > 2 (< 0.000) 1 > 3 (< 0.000)
	IT	8.95 ± 2.55	1.55 ± 0.61	3.77 ± 1.46	8.35 ± 2.25			
	FR	9.08 ± 1.56	5.03 ± 1.59	3.86 ± 0.78	8.49 ± 1.20			
LF/HF (n.u.)	SP	3.92 ± 0.54	4.27 ± 0.59	4.75 ± 0.55	3.71 ± 0.45	1.99	0.097	
	IT	4.00 ± 0.62	6.66 ± 0.72	7.83 ± 0.80	5.90 ± 0.72			
	FR	3.17 ± 0.32	4.72 ± 0.64	6.20 ± 0.59	4.14 ± 0.46			
LF (n.u.)	SP	73.94 ± 3.20	77.13 ± 2.13	78.98 ± 2.41	74.26 ± 2.89	0.92	0.447	
	IT	75.31 ± 2.43	83.85 ± 2.06	86.02 ± 1.78	81.49 ± 2.72			
	FR	75.53 ± 1.96	77.36 ± 2.81	84.09 ± 1.43	78.02 ± 1.70			
HF (n.u.)	SP	25.95 ± 3.17	22.76 ± 2.12	20.97 ± 2.40	25.66 ± 2.88	0.95	0.430	
	IT	24.65 ± 2.42	16.25 ± 2.02	13.95 ± 1.78	18.46 ± 2.72			
	FR	26.40 ± 1.94	22.79 ± 2.81	15.87 ± 1.43	21.94 ± 1.70			
SD1 (ms)	SP	28.61 ± 5.89	17.00 ± 4.77	19.08 ± 2.28	29.78 ± 5.42	0.75	0.549	
	IT	23.69 ± 4.63	8.77 ± 1.37	13.54 ± 2.23	22.15 ± 3.56			
	FR	28.80 ± 4.83	16.99 ± 3.80	15.61 ± 1.59	21.19 ± 1.43			
SD2 (ms)	SP	59.62 ± 5.78	38.23 ± 6.16	53.78 ± 3.90	67.20 ± 6.35	1.50	0.204	
	IT	58.27 ± 7.07	33.51 ± 3.97	48.54 ± 5.22	57.55 ± 5.70			
	FR	72.12 ± 6.05	47.97 ± 5.28	50.98 ± 3.75	63.28 ± 3.10			

M1: pre-defense; M2: first 1/5 of the defense; M3: last 1/5 of the defense; M4: post-defense; HRmin: minimum heart rate; HRmax: maximum heart rate; HRmean: mean heart rate; RMSSD: square root of the average of sum of the squared differences of the RR intervals; PNN50: percentage of consecutive RR intervals that differ >50 ms; LF/HF: ratio between low and high-frequency waves; LF; low-frequency wave; HF: high-frequency wave; SD1: variability of the short-term HRV; SD2: variability of the long-term HRV; SP: Spanish students; IT: Italian students; FR: French students.

**Table 2:** Changes (Mean ± SD) in subjective units of distress (SUDS), critical flicker fusion threshold before (CFFT), and the expected academic achievement in the final thesis according to different nationality.

Variable	Nationality	M1	M4	F-Value	*p*-Value
SUDS (0–100)	SP	72.63 ± 4.34	29.21 ± 5.70	1.487	0.240
	IT	72.15 ± 5.08	32.26 ± 6.40		
	FR	69.73 ± 4.03	41.84 ± 7.35		
CFFT	SP	34.10 ± 0.51	34.46 ± 0.58	1.012	0.365
	IT	35.17 ± 0.45	35.43 ± 0.67		
	FR	33.91 ± 0.85	35.02 ± 0.85		

M1: pre-defense; M4: post-defense; SP: Spanish students; IT: Italian students; FR: French students.

**Table 3:** Academic achievement in the written final thesis defense, oral final thesis defense, written + oral final thesis defense and mean academic achievement during the degree according to different nationality. Data are mean ± SD.

Variable	Nationality	Academic Achievement	F-Value	*p*-Value	Post Hoc
Written final thesis defense achievement (0–10)	SP	8.09 ± 1.23	4.00	0.041	SP > FR (0.050)
	IT	7.73 ± 2.22			
	FR	5.55 ± 3.70			
Oral final thesis defense achievement (0–10)	SP	8.80 ± 1.02	4.31	0.040	SP > FR (0.032)
	IT	7.99 ± 2.50			
	FR	5.57 ± 3.90			
Written + oral final thesis defense achievement (0–10)	SP	8.15 ± 1.22	3.66	0.063	
	IT	7.79 ± 2.63			
	FR	5.22 ± 3.46			
Mean academic achievement during the degree (0–10)	SP	7.49 ± 0.52	1.27	0.289	
	IT	7.75 ± 0.79			
	FR	7.82 ± 0.51			

SP: Spanish students; IT: Italian students; FR: French students.

**Table 4:** Expected academic achievement in the oral final thesis defense (0–10).

Variable	Nationality	M1	M4	F-Value	*p*-Value
Expected academic achievement (0–10)	SP	6.94 ± 0.15	7.18 ± 0.25	0.507	0.569
	IT	7.50 ± 0.15	7.42 ± 0.25		
	FR	6.44 ± 0.20	6.63 ± 0.20		

M1: pre-defense; M4: post-defense; SP: Spanish students; IT: Italian students; FR: French students.

## Data Availability

All the data are presented in the study.
